# Galgeun-tang Attenuates Cigarette Smoke and Lipopolysaccharide Induced Pulmonary Inflammation via IκBα/NF-κB Signaling

**DOI:** 10.3390/molecules23102489

**Published:** 2018-09-28

**Authors:** Na-Rae Shin, Chul Kim, Chang-Seob Seo, Je-Won Ko, Young-Kwon Cho, In-Sik Shin, Joong-Sun Kim

**Affiliations:** 1 College of Veterinary Medicine (BK21 Plus Project Team), Chonnam National University, 77 Yongbong-ro, Buk-gu, Gwangju 61186, Korea; tlsskfo870220@gmail.com (N.-R.S.); rheoda@gmail.com (J.-W.K.); 2 Herbal Medicinal Research Center, Korea Institute of Oriental Medicine, 1672 Yuseong-daero, Yuseong-gu, Daejeon 34054, Korea; chulnice@kiom.re.kr (C.K.); csseo0914@kiom.re.kr (C.-S.S.); 3 College of Health Sciences, Cheongju University, 298 Daesung-ro, Sangdang-gu, Cheongju-si, Chungbuk 360764, Korea; petmen@hanmail.net

**Keywords:** Galgeun-water extract, cigarette smoke, inducible nitric oxide synthase, cyclooxygenase-2, nuclear factor kappa B

## Abstract

Galgeun-tang water extract (GGWE) is used to treat various diseases such as the common cold, eczema and asthma in China and Korea. In this study, we investigated the anti-inflammatory effect of GGWE using a cigarette smoke (CS)- and lipopolysaccharide (LPS)-induced induced pulmonary inflammation mouse model. The mice were exposed to CS for a total of seven days (eight cigarettes per day for 1 h) and LPS was administered intranasally to mice on day 4. GGWE was administered by oral gavage at doses of 50 mg/kg or 100 mg/kg 1 h before exposure to CS. GGWE decreased inflammatory cell counts, and expression of inflammatory cytokines such as interleukin (IL)-6 and tumor necrosis factor alpha (TNF-α) in bronchoalveolar lavage fluid (BALF) from mice exposed to CS and LPS. GGWE reduced the expression of inducible nitric oxide synthase (iNOS) and cyclooxygenase-2 (COX-2), as well as the phosphorylation of inhibitor of kappa-B subunit alpha (IκBα) and nuclear factor kappa-B (NF-κB) in CS- and LPS-exposed mice. Histological examinations revealed that GGWE suppressed inflammatory cell infiltration into lung tissue compared to untreated CS- and LPS-exposed mice. In conclusion, GGWE effectively suppressed CS- and LPS-induced pulmonary inflammation. Our results indicate that GGWE may be used as a protective drug to control pulmonary inflammation diseases such as chronic obstructive pulmonary disease.

## 1. Introduction

Chronic obstructive pulmonary disease (COPD) is a pulmonary inflammatory disease that is mainly caused by exposure to cigarette smoke (CS) [1]. It is characterized by the destruction of normal lung architecture induced by inflammatory mediators including tumor necrosis factor alpha (TNF-α) and interleukin (IL)-6, which cause lung damage [2]. Exposure to CS induces the recruitment of inflammatory cells such as neutrophils and macrophages, which play central roles in the progression of COPD as they produce inflammatory cytokines [3]. In clinical trials, neutrophil and macrophage numbers increased in airway and parenchymal lesions of lung tissue from patients with COPD [4]. Thus, the reduction of inflammatory cell numbers is regarded as an important therapeutic strategy in CS- induced pulmonary inflammatory disease.

Nuclear factor kappa-B (NF-κB) is a ubiquitous transcription factor complex that is activated when dissociating from inhibitor of kappa-B subunit alpha (IκBα) after IκBα phosphorylation, which is catalyzed by IκB kinase [5,6]. Activated NF-κB translocates to the nucleus, and it eventually produces several inflammatory mediators. NF-κB activation is therefore closely associated with the progression of COPD [6]. NF-κB-mediated inducible nitric oxide synthase (iNOS) and cyclooxygenase-2 (COX-2) are considered to be particularly important mediators in the development and aggravation of pulmonary inflammation induced by CS [7,8,9]. Furthermore, the expression of iNOS and COX-2 is increased by activated NF-κB through the phosphorylation of IκBα in CS condensate-stimulated cells [9]. In clinical trials, iNOS and COX-2 expression is elevated in smokers compared to non-smokers [7,8]. Thus, the reduction of iNOS and COX-2 expression is associated with a reduction in CS-induced pulmonary inflammation, and it is regarded as a target for the discovery of novel therapeutic agents for the treatment of pulmonary inflammatory disease.

We searched electronic databases for relevant studies published before August 2018 in the Oriental Medicine Advanced Searching Integrated System (OASIS). According to the OASIS, Galgeun-tang water extract (GGWE) is a traditional oriental herbal remedy that is known as Gegen-tang in China and Kakkon-to in Japan. It is composed a mixture of seven herbal preparations (Puerariae Radix, Cinnamomi Ramulus, Ephedrae Herba, Paeoniae Radix, Glycyrrhizae Radix et Rhizoma, Zingiberis Rhizoma Recens, and Zizyphi Fructus). This mixture has long been used to treat various diseases, especially respiratory diseases [10,11]. A previous study demonstrated that GGWE attenuated inflammatory responses in lipopolysaccharide (LPS)-stimulated BV-2 microglial cells via inhibition of the NF-κB pathway [12]. In another study, GGWE exhibited a strong free radical scavenging activity [13]. In mouse studies, GGWE exhibited therapeutic efficacy in influenza virus infected mice. [14,15]. In clinical patients, the effects of GGWE have been reported to be effective in chronic sinusitis, nasal obstruction, pneumonia, allergic rhinitis, and pneumonia, including common colds [10]. However, there are no studies available regarding the effect of GGWE on CS- and LPS-induced pulmonary inflammation.

The objective of our study was to determine whether GGWE is effective in reducing CS- and LPS-induced pulmonary inflammation. In order to evaluate the anti-inflammatory effect of GGWE, we recorded inflammatory cell counts and levels of inflammatory cytokines, as well as measured the expression of inflammatory proteins, including iNOS, COX-2, IκBα, and NF-κB in a mouse model of CS- and LPS-induced pulmonary inflammation.

## 2. Results

### 2.1. HPLC Analysis of GGWE

**Figure 1:**
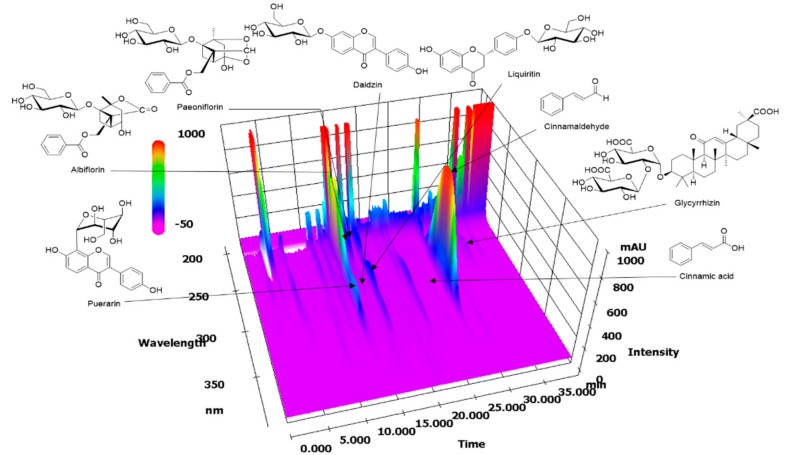
Three-dimensional chromatogram of GGWE by HPLC-PDA.

The simultaneous determination of eight marker components in Galgeun-tang was performed using an optimized HPLC method at 230 nm (albiflorin and paeoniflorin), 254 nm (puerarin, daidzin, and glycyrrhizin), and 280 nm (liquiritin, cinnamic acid, and cinnamaldehyde). As a result, the eight analytes eluted within 35 min and the individual retention times were 13.790, 15.0118, 15.695, 15.922, 17.636, 25.802, 28.109, and 34.109 min for puerarin, albiflorin, daidzin, paeoniflorin, liquiritin, cinnamic acid, cinnamaldehyde, and glycyrrhizin, respectively (Figure 1). The amounts of these components in GGWE were 10.31, 2.53, 2.04, 3.48, 7.06, 2.04, 7.50, and 12.38 mg/g, respectively.

### 2.2. GGWE Decreased CS and LPS Induced Inflammatory Cell Recruitment

**Figure 2:**
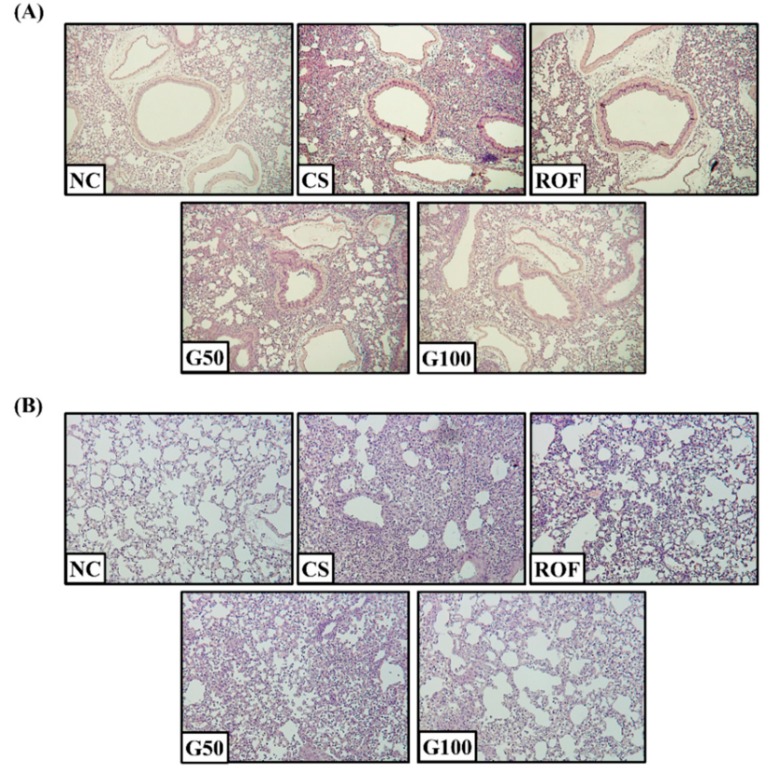
GGWE attenuate inflammatory cell infiltration in H&E staining. (**A**) Peribronchial region, (**B**) alveolar region. NC, non-treated mice; CS, CS and LPS exposure mice; ROF, roflumilast 10 mg/kg and CS and LPS exposure mice; G50, GGWE 50 mg/kg and CS and LPS exposure mice; G100, GGWE 100 mg/kg and CS and LPS exposure mice.

Lung tissue from mice in the CS group showed extensive inflammatory cell infiltration into the peribronchial and alveolar lesions compared to those of the NC group (Figure 2A,B). The roflumilast-treated group showed a decrease in inflammatory cell infiltration into the lung tissue, compared with the CS group. In addition, GGWE-treated groups showed reduced inflammatory cell infiltration into the lung tissue, compared with the CS group. Notably, the group treated with a high dose of GGWE showed similar results to the roflumilast-treated group.

**Figure 3:**
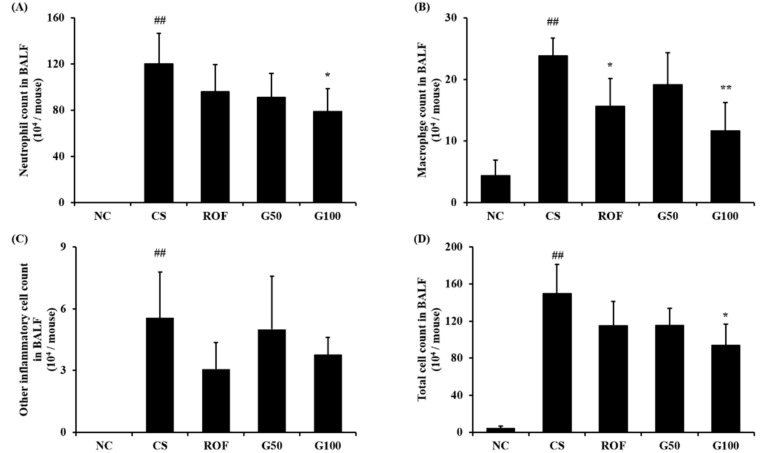
GGWE reduce inflammatory cell counts in bronchoalveolar lavage fluid (BALF). (**A**) number of neutrophils, (**B**) number of macrophages, (**C**) number of other cells and (**D**) number of total cells. NC, non-treated mice; CS, CS and LPS exposure mice; ROF, roflumilast 10 mg/kg and CS and LPS exposure mice; G50, GGWE 50 mg/kg and CS and LPS exposure mice; G100, GGWE 100 mg/kg and CS and LPS exposure mice. The values shown are the mean ± SD. ## *p* < 0.01 vs. NC; # *p* < 0.05 vs. NC; ** *p* < 0.01 vs. CS; * *p* < 0.05 vs. CS.

Bronchoalveolar lavage fluid (BALF) analysis revealed that the number of inflammatory cells in the CS group increased in comparison to that in the NC group (Figure 3). In particular, the number of neutrophils and macrophage was markedly elevated in the CS group compared to that in the NC group. The roflumilast-treated group showed a significant decrease in inflammatory cell counts in comparison to those of the CS group. GGWE-treated groups also showed decreased inflammatory cell counts compared with the CS group. A significant reduction in the number of neutrophils, macrophages, and total cells in the high-dose GGWE-treated group was observed compared with that in the CS group.

### 2.3. GGWE Reduced CS and LPS Induced Inflammatory Mediators in BALF

**Figure 4:**
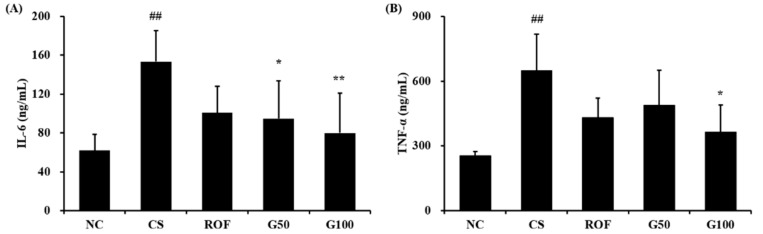
GGWE decrease the production of inflammatory cytokines. (**A**) IL-6 and (**B**) TNF-α. NC, non-treated mice; CS, CS and LPS exposure mice; ROF, roflumilast 10 mg/kg and CS and LPS exposure mice; G50, GGWE 50 mg/kg and CS and LPS exposure mice; G100, GGWE 100 mg/kg and CS and LPS exposure mice. The values shown are the mean ± SD. ## *p* < 0.01 vs. NC; # *p* < 0.05 vs. NC; ** *p* < 0.01 vs. CS; * *p* < 0.05 vs. CS.

CS group markedly increased inflammatory mediators, including IL-6 and TNF-α, in comparison to NC group (NC group; IL-6: 61.78 ± 16.99 TNF-α: 254.62 ± 17.93, CS group; IL-6: 153.49 ± 31.76 TNF-α: 648.7 ± 168.64, Figure 4A,B, respectively). The roflumilast-treated group had decreased levels of IL-6 and TNF-α compared with those of the CS group (ROF group; IL-6: 100.6 ± 27.3 TNF-α: 429.72 ± 91.55). GGWE-treated groups reduced the levels of IL-6 and TNF-α in comparison to those of CS group (G50 group; IL-6: 94.83 ± 38.73 TNF-α: 487.62 ± 163.05, G100; IL-6: 79.93 ± 40.75 TNF-α: 363.15 ± 126.69). In particular, a high dose in the GGWE-treated group showed a significant reduction compared with those in the CS group.

### 2.4. GGWE Decreased CS- and LPS-Induced Expression of iNOS and COX-2

**Figure 5:**
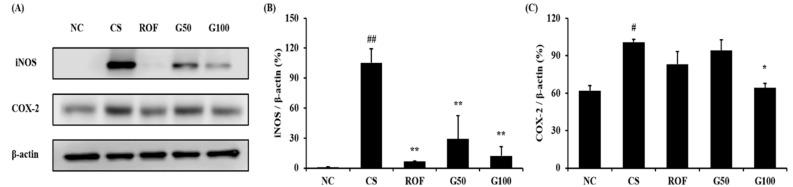
GGWE reduced the expression of iNOS and COX-2 in lung tissue. (**A**) Expression of iNOS and COX-2, (**B**) quantitative analysis for iNOS expression, (**C**) quantitative analysis for COX-2 expression. NC, non-treated mice; CS, CS and LPS exposure mice; ROF, roflumilast 10 mg/kg and CS and LPS exposure mice; G50, GGWE 50 mg/kg and CS and LPS exposure mice; G100, GGWE 100 mg/kg and CS and LPS exposure mice. The values shown are the mean ± SD. ## *p* < 0.01 vs. NC; # *p* < 0.05 vs. NC; ** *p* < 0.01 vs. CS; * *p* < 0.05 vs. CS.

**Figure 6:**
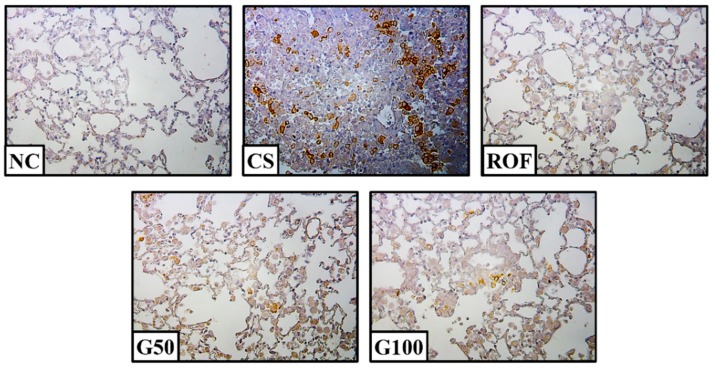
GGWE attenuate the activity of iNOS in lung tissue. NC, non-treated mice; CS, CS and LPS exposure mice; ROF, roflumilast 10 mg/kg and CS and LPS exposure mice; G50, GGWE 50 mg/kg and CS and LPS exposure mice; G100, GGWE 100 mg/kg and CS and LPS exposure mice.

Lung tissues from the CS group showed increased iNOS expression compared to those of the NC group, while iNOS expression was significantly reduced in the ROF group compared to that in the CS group (Figure 5A,B). iNOS expression was also decreased in the GGWE-treated group compared to that in the CS group. These results were consistent with immunohistochemistry results (Figure 6). Consistent with iNOS expression, COX-2 expression increased significantly in the CS group in comparison to that in the NC group (Figure 5A,C). The GGWE-treated groups showed reduced COX-2 expression in comparison to the CS group.

In particular, the high-dose GGWE-treated group showed a significant reduction in COX-2 expression.

### 2.5. GGWE Reduced Phosphorylation of IκBα and NF-κB

**Figure 7:**
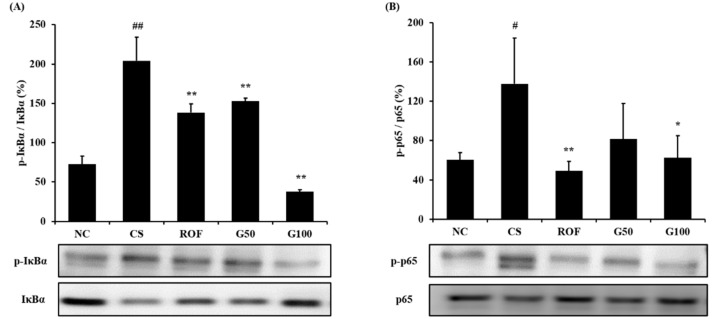
GGWE decreased the phosphorylation of IκBα and NF-κB expression in lung tissue. (**A**) phosphorylation of IκBα expression and quantitative analysis for phosphorylation of IκBα expression, (**B**) phosphorylation of NF-κB expression and quantitative analysis for the phosphorylation of NF-κB expression. NC, non-treated mice; CS, CS and LPS exposure mice; ROF, roflumilast 10 mg/kg and CS and LPS exposure mice; G50, GGWE 50 mg/kg and CS and LPS exposure mice; G100, GGWE 100 mg/kg and CS and LPS exposure mice. The values shown are the mean ± SD. ## *p* < 0.01 vs. NC; # *p* < 0.05 vs. NC; ** *p* < 0.01 vs. CS; * *p* < 0.05 vs. CS.

IκBα phosphorylation in lung tissue was markedly elevated in the CS group, compared to that in the NC group (Figure 7A). The roflumilast-treated group showed reduced CS- and LPS-induced IκBα phosphorylation. Furthermore, CS- and LPS-induced IκBα phosphorylation was also decreased in the GGWE-treated group, and even more so in the high-dose GGWE-treated group. Consistently, the CS group showed markedly increased phosphorylation of NF-κB in comparison to the NC group (Figure 7B). By contrast, phosphorylation of NF-κB was reduced in the GGWE-treated groups in comparison to that in the CS group. In particular, the high-dose GGWE-treated group showed a significant reduction.

## 3. Discussion

COPD is now the fifth leading cause of death worldwide, and it afflicts 15–17 million people in the United States alone [16]. Although many drugs have been developed and are used to treat COPD, the use of such drugs is limited because of their associated side effects [17]. There is therefore a growing need for the development of effective new drugs without side effects. In this study, we investigated the effects of GGWE on CS- and LPS-induced pulmonary inflammation. GGWE significantly reduced CS- and LPS-induced inflammatory cell counts in BALF and inflammatory cell infiltration in lung tissues, which was accompanied by a reduction in IL-6, TNF-α, iNOS, and COX-2 expression. Furthermore, GGWE suppressed the CS- and LPS-induced increase in IκBα and NF-κB phosphorylation.

In COPD, the activation of NF-κB is mediated through the phosphorylation of IκBα and regulates the production of inflammatory mediators, such as cytokines and chemokines, resulting in the development and aggravation of inflammatory responses in damaged lesions [6]. In particular, macrophages and neutrophils are recruited via these events, and they play important roles in COPD though the release of pro-inflammatory mediators, and the activation of other inflammatory signaling cascades [3]. Here, GGWE significantly decreased CS- and LPS-induced inflammatory cell numbers in BALF. This result was consistent with those of histological examinations. GGWE suppressed inflammatory cell infiltration into lung tissue in CS- and LPS-exposed animals. In addition, the phosphorylation of NF-κB was reduced in GGWE-treated groups, compared to that in the CS group. As mentioned above, the activation of NF-κB produces pro-inflammatory mediators such as IL-6 and TNF-α via translocation to the nucleus [3]. In this study, GGWE-treated groups showed a significant reduction in IL-6 and TNF-α expression in BALF from CS- and LPS-exposed animals. These results indicate that GGWE possesses anti-inflammatory properties, and it reduces CS- and LPS-induced pulmonary inflammation via the inhibition of NF-κB activation.

iNOS and COX-2 expression is involved in the activation of NF-κB, and it promotes the production of various inflammatory mediators, as well as the recruitment of inflammatory cells [18]. In particular, overexpression of iNOS and COX-2, as well as activation of NF-κB, was observed in patients with COPD [19,20]. These responses were observed in experimental models. The expression of iNOS and COX-2 markedly increased in CS-exposed mice and cigarette smoke extract (CSE)-stimulated cells, which was also accompanied by the activation of NF-κB [21,22]. Thus, suppression of iNOS and COX-2 expression may be used as a therapeutic index indicating a decrease in CS-induced inflammation. In this study, GGWE-treated groups showed a significant reduction in the expression of iNOS and COX-2 in lung tissue, compared with the CS group. These results indicate that GGWE has the potential to control CS- and LPS-induced pulmonary inflammation via the suppression of iNOS and COX-2 expression, and inhibition of NF-κB activation.

GGWE is a traditional herbal decoction that has been used to treat common cold, fevers, and inflammatory diseases in China, Japan, and Korea [10,23]. Experimental studies have revealed that GGWE has anti-viral, anti-allergic, antioxidant, and antiadipogenic effects [13,14,15,24,25]. Furthermore, GGWE was shown to reduce inflammatory mediators such as inflammatory cytokines, iNOS, and NF-κB expression through the inhibition of NF-κB in LPS-stimulated microglial cells [12]. GGWE also attenuated atopic dermatitis induced by dust mite extract via the reduction of inflammatory mediators such as NO and TNF-α [18]. These reports are consistent with results from this study. Furthermore, GGWE contains various active components, which are considered to be closely related with the anti-inflammatory effects observed in this study. A previous report revealed that GGWE contains known components including cinnamic acid, puerarin, paeoniflorin, and glycyrrhizin, and this was confirmed in our experiments [11]. Cinnamic acid was shown to decrease the production of inflammatory mediators such as NO, prostaglandin E2, IL-6, and TNF-α in LPS-stimulated RAW264.7 cells [26]. Puerarin and glycyrrhizin inhibited inflammatory responses in an LPS-induced acute lung injury model and an imiquimod-induced psoriasis-like skin model, respectively [27,28]. In addition, paeoniflorin has shown anti-inflammatory properties in various experimental models such as collagen-induced rheumatoid arthritis, high-fat diet-induced atherosclerosis, and LPS-induced acute lung injury [29,30,31]. The effects of these four components is closely associated with the inactivation of NF-κB. These previous studies strongly support the anti-inflammatory effects of GGWE observed in our study.

In conclusion, we evaluated the therapeutic effects of GGWE on CS- and LPS-induced pulmonary inflammation. In this study, GGWE decreased CS- and LPS-induced elevated inflammatory cell counts, and expression of inflammatory cytokines and proteins. These results were associated with the suppression of NF-κB activation in lung tissues. Therefore, our study suggests that GGWE has the potential to treat CS-induced pulmonary inflammatory diseases.

## 4. Materials and Methods

### 4.1. Chemicals and Reagents

Eight reference standards (albiflorin, cinnamaldehyde, cinnamic acid, daidzin, glycyrrhizin, liquiritin, paeoniflorin, and puerarin) were purchased from Wako (Osaka, Japan) and the purities of these reference standards were greater than 98.0%, as evaluated via high-performance liquid chromatography (HPLC). The HPLC-grade reagents, methanol, acetonitrile, and water were obtained from J.T. Baker (Phillipsburg, NJ, USA). Analytical-grade glacial acetic acid was procured from Junsei (Tokyo, Japan).

### 4.2. Plant Materials

The seven raw herbal medicines (Puerariae Radix, Cinnamomi Ramulus, Ephedrae Herba, Radix Paeoniae, Glycyrrhizae Radix et Rhizoma, Zingiberis Rhizoma Recens, and Zizyphi Fructus) composing Galgeun-tang were purchased from Omniherb (Yeongcheon, Korea) and HMAX (Jecheon, Korea) in February 2008. The identity of each raw material was verified by Prof. Je-Hyun Lee, Oriental Medicine, Dongguk University (Gyeongju, Korea) and Prof. Young-Bae Seo, Oriental Medicine, Daejeon University (Daejeon, Korea). Voucher specimens (2008-KE02-1 to KE02-7) were deposited at the K-herb Research Center, Korea Institute of Oriental Medicine (KIOM, Daejeon, Korea).

### 4.3. Preparation of GGWE

GGWE was prepared at KIOM. Briefly, the seven component herbs of GGWE, Puerariae Radix (717 g), Cinnamomi Ramulus (477 g), Ephedrae Herba (477 g), Paeoniae Radix (477 g), Glycyrrhizae Radix et Rhizoma (477 g), Zingiberis Rhizoma Recens (477 g), and Zizyphi Fructus (398 g), were mixed and extracted in a 10-fold volume (35 L) of water at 100 °C for 2 h under pressure (98 kPa) using an electric extractor (COSMOS-660; Kyungseo Machine Co., Incheon, Korea). The water extract solution was evaporated to dryness and freeze-dried to yield a powder (extract amount: 441.6 g, yield: 12.6%).

### 4.4. HPLC Analysis of GGWE

Stock solutions of eight reference standards were dissolved in methanol at a concentration of 1000.0 μg/mL and stored at 4 °C. For the HPLC analysis of Galgeun-tang, a Prominence LC-20A HPLC system (Shimadzu Co., Kyoto, Japan), consisting of a solvent delivery unit, an on-line degasser, a column oven, an autosampler, and a photodiode array detector was used. The data were processed using LC solution software (Version 1.24, SP1). The analytical column used was a Gemini C18 (250 × 4.6 mm; particle size 5 μm, Phenomenex, Torrance, CA, USA) and it was maintained at 40 °C. The mobile phases consisted of 1.0% (*v/v*) acetic acid in distilled water (solvent A) and 1.0% (*v/v*) acetic acid in acetonitrile (solvent B). The gradient used was as follows: 5–70% B, 0–40 min; 70–100% B, 40–45 min; 100% B, 45–50 min; and 100–5% B, 55 min. The flow-rate was 1.0 mL/min and the injection volume was 10 μL.

### 4.5. Experimental Design

Six–eight-week old C57BL/6 mice were purchased from the Samtako Co. (Osan, Korea). The Institutional Animal Care and Use Committee of Chonnam National University approved the protocols for the animal study (CNU IACUC-YB-R-2016-18), and the animals were cared for in accordance with the Guidelines for Animal Experiments of Chonnam National University. The experiment model, mice administrated LPS (5 ug per mouse in 50 uL PBS) at day 5, and CS exposure was performed eight cigarettes per day, 1 h for seven days using cigarette smoke generator (Daehan Biolink, Korea) in a chamber. The cigarettes used were 3R4F research cigarettes, containing 0.76 mg nicotine and 9.4 mg tar. There were five mice in each of the following groups: NC, mice receiving no CS exposure or treatment; CS: mice receiving CS exposure and LPS injection; ROF: mice receiving CS exposure, LPS injection, and 10 mg/kg roflumilast treatment; G50: mice receiving CS exposure, LPS injection, and 50 mg/kg GGWE treatment; G100, mice receiving CS exposure, LPS injection, and 100 mg/kg GGWE treatment.

### 4.6. Collection of Bronchoalveolar Lavage Fluid (BALF)

BALF collection was performed by infusing two sequential aliquots of 0.7 mL ice-cold PBS into the mice’s lungs using a tracheal cannula. A total of 1.4 mL BALF was centrifuged at 1500 rpm for 5 min at 4 °C. The supernatant was collected and stored at −80 °C. The pellets were centrifuged on slides using a Cytospin (Hanil Science Industrial, Seoul, Korea), and they were stained with Diff-Quik^®^ (Sysmex, Kobe, Japan). The stained cells were used to record differential cell counts. Total inflammatory cell counts were determined using a cell counter (Countess II, Thermo Fisher Scientific, San Diego, CA, USA). Pro-inflammatory cytokine expression was measured in BALF supernatants using enzyme-linked immunosorbent assay (ELISA) kits (R&D system, Minneapolis, MN, USA), according to the manufacturer’s protocols. The amount of pro-inflammatory cytokines was calculated according to the measured absorbance using the standard curve.

### 4.7. Western Blot

The lung tissues were homogenized and protein concentration was determined using a Bradford reagent assay (Bio-Rad, Hercules, CA, USA). The proteins were separated via 10% sodium dodecyl sulfate polyacrylamide gel electrophoresis (SDS-PAGE) and transferred to polyvinylidene fluoride (PVDF) membranes. The membranes were then incubated with 5% skim milk blocking solution for 1 h. After incubation, the membranes were blotted with primary antibody at 4 °C overnight. Primary antibodies used were as follows: COX2 (Abcam, Cambridge, UK), iNOS (Abcam), phospho (p)-IκBα (Cell Signaling, Danvers, MA, USA), IκBα (Cell Signaling), p-p65 (Abcam), p65 (Abcam), and β-actin (Cell Signaling). The membranes were incubated with horseradish peroxidase (HRP)-conjugated secondary goat immunoglobulin G (IgG; Jackson Immuno Research, West Grove, PA, USA) for 1 h. The bands were visualized using an electrochemiluminescence (ECL) kit (Thermo Fisher Scientific) and ChemiDoc (Bio-Rad). Quantitative analysis of protein expression was conducted using IMT i-Solution software (IMT i-Solution Inc., Vancouver, BC, Canada).

### 4.8. Histopathological Examination

The lungs of mice were fixed using a 10% buffered formalin solution for three days at room temperature. Fixed lung tissues were sectioned (4 μm thickness), deparaffinized using xylene, and dehydrated using ethanol. Following a 5-min wash with distilled water, tissue sections were stained using hematoxylin and eosin (H&E) stain to evaluate inflammatory cell infiltration.

To perform immunohistochemical analysis, deparaffinized and dehydrated tissues were washed with PBS-T (PBS containing 0.05% Tween 20) and incubated for 20 min with goat serum at room temperature to block nonspecific protein binding. The tissues were incubated with primary mouse anti-rabbit iNOS antibody (diluted 1:100, Abcam) for 2 h at room temperature, washed three times, and incubated with biotinylated secondary antibody for 1 h at room temperature. Tissues were then incubated with an avidin-biotin-peroxidase complex (Vector Laboratories, Burlingame, CA, USA) for 1 h at room temperature and further incubated with diaminobenzidine (DAB, Abcam) for 5 min before visualizing using a microscope.

### 4.9. Statistical Analysis

The data are expressed as means ± standard deviation. Data were analyzed via an analysis of variance (ANOVA), followed by Dunnett’s multiple comparison testing. A value of *p* < 0.05 indicated a statistically significant difference.
